# Publisher Correction: Influences of organic carbon speciation on hyporheic corridor biogeochemistry and microbial ecology

**DOI:** 10.1038/s41467-018-03572-7

**Published:** 2018-03-07

**Authors:** James C. Stegen, Tim Johnson, James K. Fredrickson, Michael J. Wilkins, Allan E. Konopka, William C. Nelson, Evan V. Arntzen, William B. Chrisler, Rosalie K. Chu, Sarah J. Fansler, Emily B. Graham, David W. Kennedy, Charles T. Resch, Malak Tfaily, John Zachara

**Affiliations:** 1 Pacific Northwest National Laboratory, Richland, WA 99352 USA; 2 Department of Microbiology, The Ohio State University, Columbus, OH 43210 USA; 3 School of Earth Sciences, The Ohio State University, Columbus, OH 43210 USA

Correction to: *Nature Communications* 10.1038/s41467-018-02922-9, published online 08 February 2018

The original version of this Article contained an error in Fig. 6e, in which the text in the legend was omitted. This has been corrected in both the PDF and HTML versions of the article.

