# Supplementary material for: Influences of organic carbon speciation on hyporheic corridor biogeochemistry and microbial ecology
Source: Nat Commun. 2018 Feb 8;9:585. doi: 10.1038/s41467-018-02922-9 (PMC5805721; doi:10.1038/s41467-018-02922-9)
Supplement: Supplementary file 1 — Supplementary Information [file 41467_2018_2922_MOESM1_ESM.pdf]

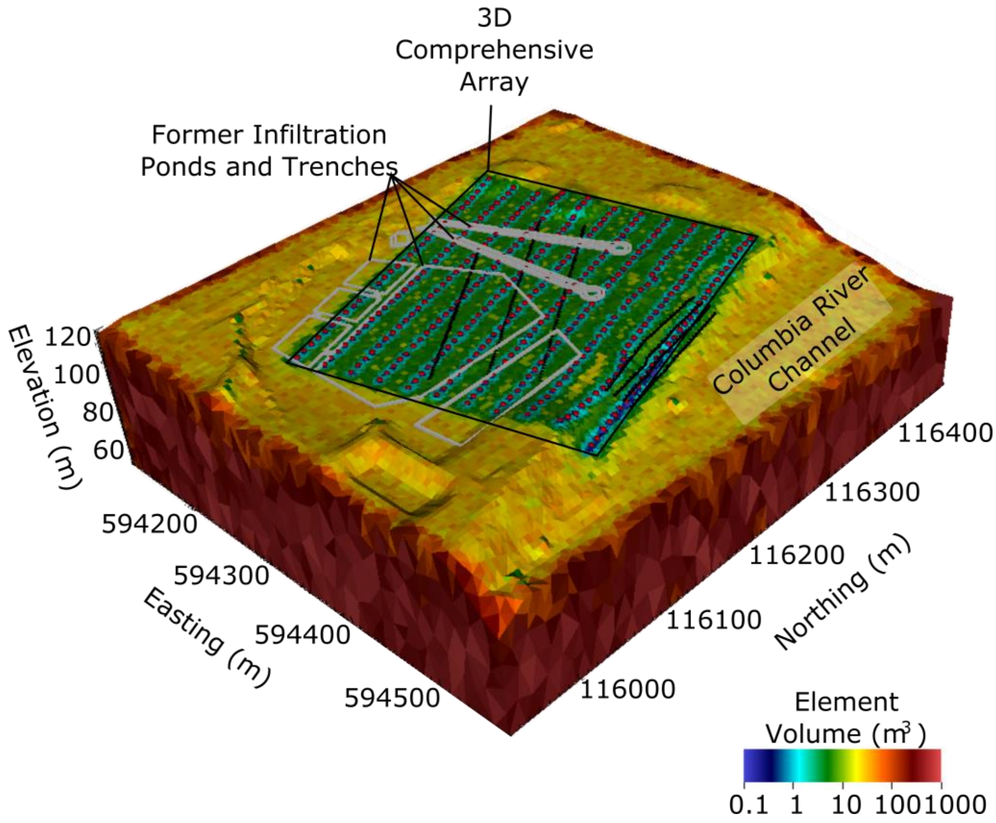

Supplementary Figure 1. ERT computational mesh. The computational mesh shows electrode locations (red dots), surface topography, and riverbed bathymetry local to the electrode array. Modified from Johnson et al.[1]. The electrodes are the same as those shown in Figure 2 in the main manuscript, but note the change in orientation.

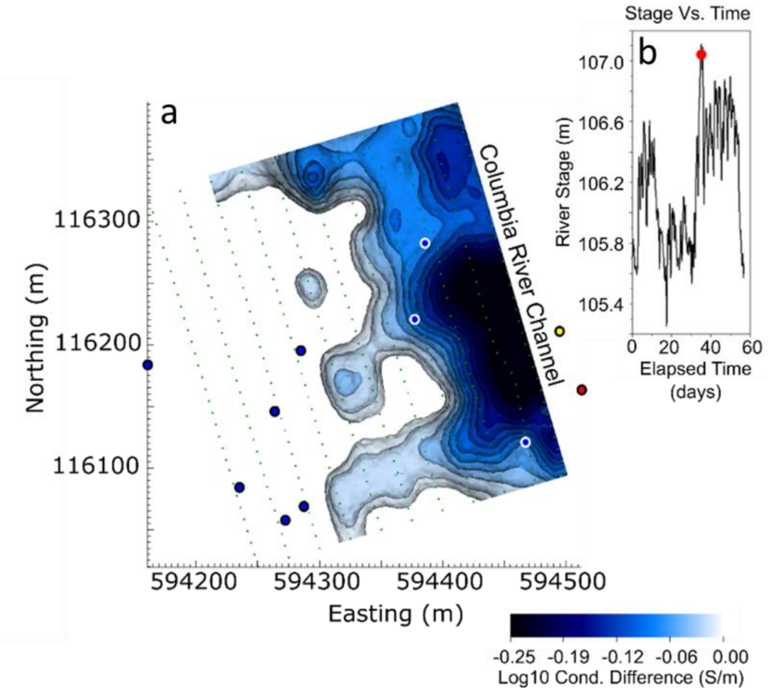

Supplementary Figure 2. Spatial projection of electrical conductivity (EC) shift relative to a 100% groundwater baseline condition. (a) Plan view ERT image of the change in EC caused by river water intrusion at peak stage. Changes in EC are shown as 3D isosurfaces. Colored circles indicate sampling locations within groundwater wells (blue circles with black or white border), the nearshore hyporheic zone (yellow with black border), and the river water sampling location (red with black border). The nearshore and river water sampling locations were within the river channel (see Figure 2 in the main manuscript for a complete view). Small green dots are the ERT electrodes. (b) River stage during the monitoring period, and the red circle at peak stage indicates the point in time shown in panel (a). Modified from Johnson et al.[1].

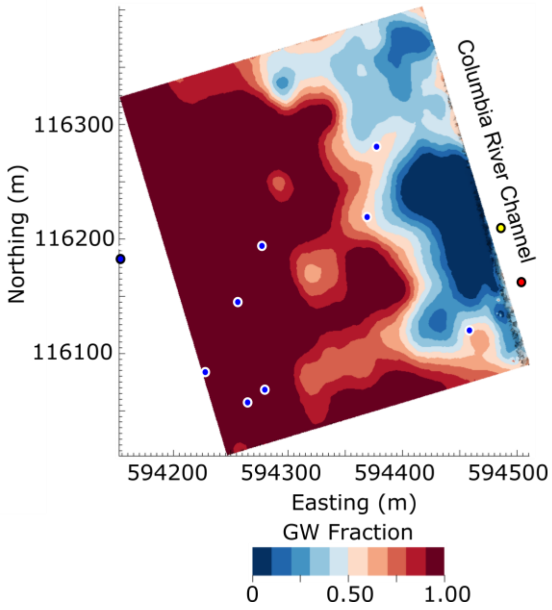

Supplementary Figure 3. ERT estimated groundwater (GW) fraction at peak stage shown in Supplementary Figure 2. The conversion to groundwater fraction was done using equations 4 and 5 (see the Methods section in the main manuscript). Modified from Johnson et al.[1]

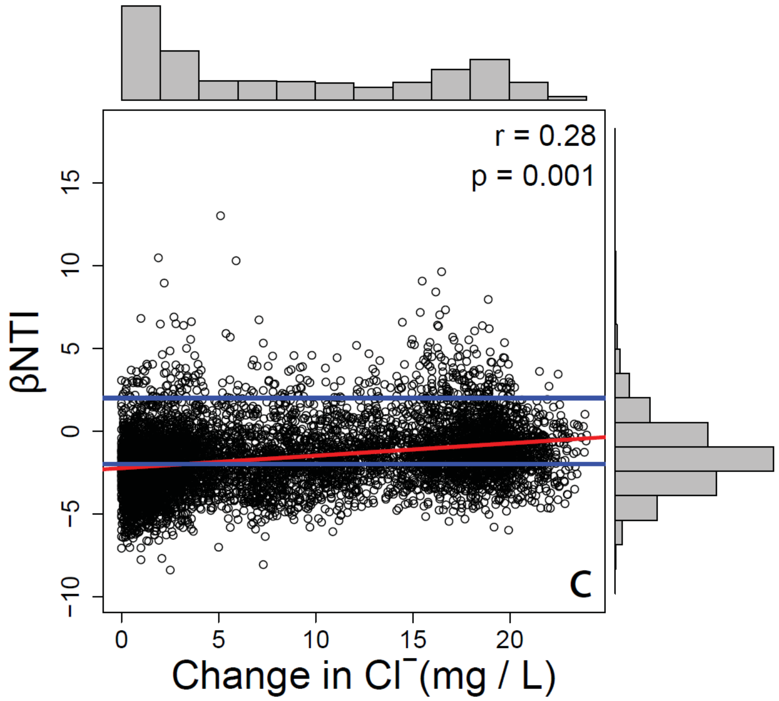

Supplementary Figure 4. Ecological responses of microbial community composition to changes in groundwater-surface water mixing. Panels show the relationship between βNTI and changes in Cl^- concentration for samples collected from the nearshore hyporheic zone and the groundwater aquifer. The concentration of Cl^- is used as a conservative tracer of groundwater-surface water mixing, which was previously validated in the study system[2]. βNTI measures the deviation between observed and expected turnover in microbial community composition, where the expected turnover is based on stochastic community assembly. βNTI therefore provides a measure of the degree to which turnover in community composition (through space or time) is governed by deterministic ecological selection. This use and interpretation of βNTI has been previously validated[3]. The red line represent the linear regression model and blue lines indicate significance thresholds for βNTI, whereby |βNTI| > 2 is considered a significant deviation from the stochastic expectation[3,4]. The patterns indicate that changes in groundwater-surface water mixing do not strongly alter the ecological selective environment. A strong shift in the selective environment would be indicated by βNTI < -2 when the change in Cl^- is near 0 and βNTI > +2 when the change in Cl^- is maximized[4]. Instead, when the change in Cl^- is maximized, βNTI is between -2 and +2, which indicates that changes in the selective environment (in response to changes in groundwater-surface water mixing conditions) are not sufficient to deterministically drive shifts in community composition.

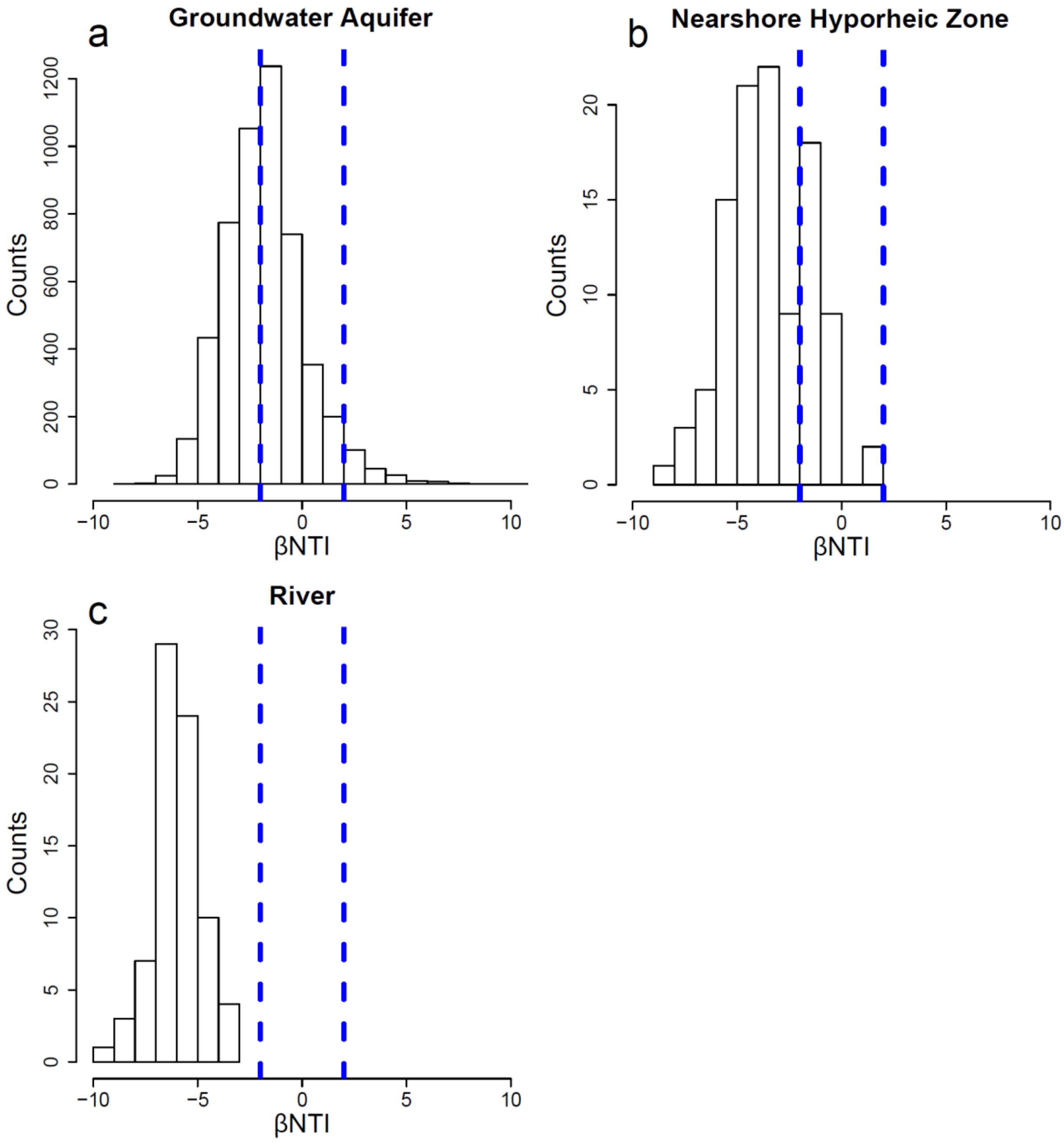

Supplementary Figure 5. Ecological null model outputs for microbial community turnover. Panels show histograms of βNTI within (a) the groundwater aquifer, (b) the nearshore hyporheic zone, and (c) the river. Dashed blue lines indicate significance thresholds of -2 and +2. The majority of βNTI values fall below -2, which indicates that community composition was held relatively constant due to ecological selective pressures being consistent through space and time within each system compartment. This situation has been referred to as homogeneous selection[3,4].

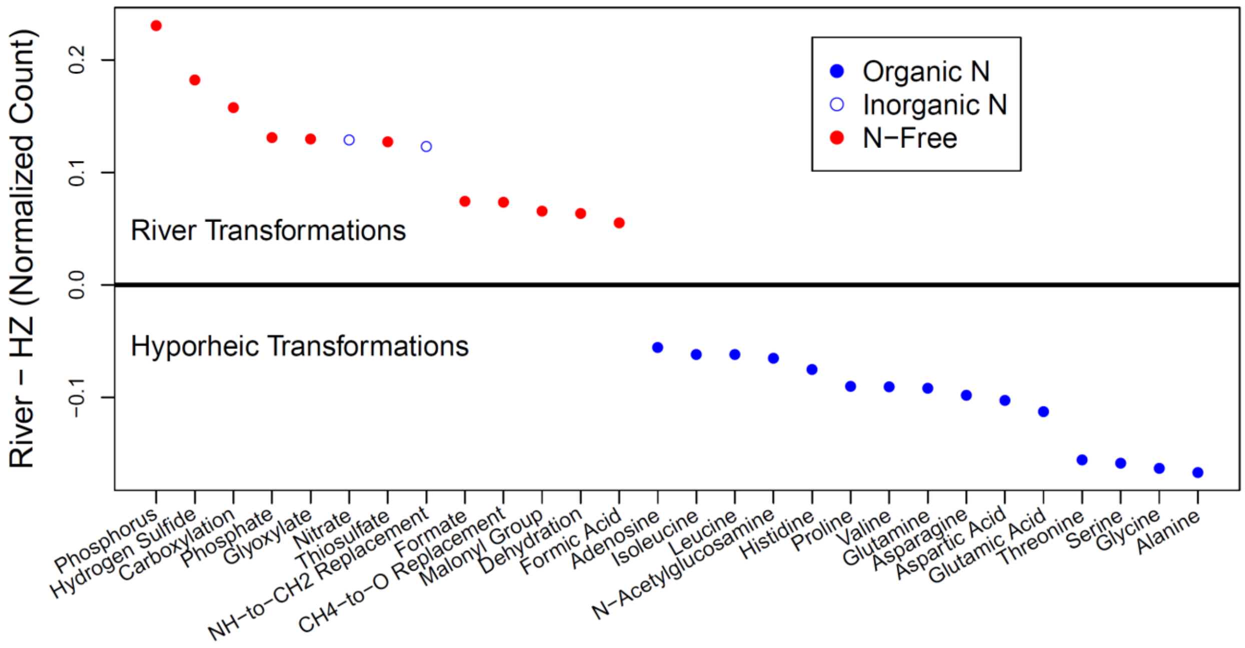

Supplementary Figure 6. Biochemical transformations inferred from FTICR-MS data that appear to be overrepresented in either the river or the nearshore hyporheic zone (HZ). Values along the vertical axis were derived from the same analysis used to generate Figure 6e in the main manuscript. The difference between the two figures is that here all peaks were retained in the HZ and river FTICR-MS datasets. Analysis outcomes shown in Figure 6e were based on first dropping the FTICR-MS peaks shared between the two datasets. While there are some quantitative differences between the two analyses, both show the same qualitative patterns and lead to the same conceptual inferences. Similar to Figure 6e, for clarity, only transformations with an absolute value on the vertical axis of 0.05 are shown (see Supplementary Data 2). Values of 0 on the vertical axis indicate equal representation, while values above/below 0 indicate overrepresentation in the river/HZ, respectively. Colors indicate whether a given transformation involves N, and the open/closed blue symbols indicate whether the N involved in the transformation is inorganic (e.g., nitrate) or organic (e.g., amino acids).

Supplementary References

1 Johnson, T. *et al.* Four-dimensional electrical conductivity monitoring of stage-driven river water intrusion: Accounting for water table effects using a transient mesh boundary and conditional inversion constraints. *Water Resour Res* **51**, 6177-6196, doi:10.1002/2014WR016129 (2015).

2 Stegen, J. C. *et al.* Groundwater-surface water mixing shifts ecological assembly processes and stimulates organic carbon turnover. *Nat Commun* **7**, Artn 11237, doi:10.1038/Ncomms11237 (2016).

3 Stegen, J. C., Lin, X., Fredrickson, J. K. & Konopka, A. Estimating and mapping ecological processes influencing microbial community assembly. *Front. Microbiol.* **6**, 370, doi:310.3389/fmicb.2015.00370 (2015).

4 Dini-Andreote, F., Stegen, J. C., van Elsas, J. D. & Salles, J. F. Disentangling mechanisms that mediate the balance between stochastic and deterministic processes in microbial succession. *P Natl Acad Sci USA* **112**, E1326-E1332, doi:10.1073/pnas.1414261112 (2015).
