# Supplementary material for: Influences of organic carbon speciation on hyporheic corridor biogeochemistry and microbial ecology
Source: Nat Commun. 2018 Feb 8;9:585. doi: 10.1038/s41467-018-02922-9 (PMC5805721; doi:10.1038/s41467-018-02922-9)
Supplement: Supplementary file 3 — Description of Additional Supplementary Files [file 41467_2018_2922_MOESM3_ESM.pdf]

Description of Additional Supplementary Files

File Name: Supplementary Data 1
Description: Summary of biochemical transformation analyses inferred from FTICR-MS data using peaks unique to the river or hyporheic zone datasets.

File Name: Supplementary Data 2
Description: Summary of biochemical transformation analyses inferred from FTICR-MS data using all peaks in the river or hyporheic zone datasets.

File Name: Supplementary Data 3
Description: Aqueous data used in mixing model analyses, and Gibbs free energy analyses.
